# Therapeutic Potential of Novel Silver Carbonate Nanostructures in Wound Healing and Antibacterial Activity Against *Pseudomonas chengduensis* and *Staphylococcus aureus*

**DOI:** 10.3390/ph17111471

**Published:** 2024-11-01

**Authors:** Tehmina Khan, Ali Umar, Zakia Subhan, Muhammad Saleem Khan, Hafeeza Zafar Ali, Hayat Ullah, Sabeen Sabri, Muhammad Wajid, Rashid Iqbal, Mashooq Ahmad Bhat, Hamid Ali

**Affiliations:** 1 Department of Zoology, Faculty of Life Sciences, University of Okara, Okara 56130, Pakistanaumar2102@gmail.com (A.U.);; 2 Department of Pharmacology, Institute of Medical Science (KMU-IMS), Khyber Medical University, Kohat 26000, Pakistan; 3 Institute of Pharmaceutical Sciences, Khyber Medical University, Peshawar 25000, Pakistan; 4 Department of Chemistry, University of Okara, Okara 56130, Pakistan; 5 Department of Microbiology and Molecular Genetics, Faculty of Life Sciences, University of Okara, Okara 56130, Pakistan; 6 Department of Agronomy, Faculty of Agriculture and Environment, The Islamia University of Bahawalpur, Bahawalpur 63100, Pakistan; rashid.iqbal@iub.edu.pk; 7 Department of Life Sciences, Western Caspian University, Baku AZ1001, Azerbaijan; 8 Department of Pharmaceutical Chemistry, College of Pharmacy, King Saud University, Riyadh 11451, Saudi Arabia; mabhat@ksu.edu.sa; 9 School of Material Science and Engineering, Tiangong University, Tianjin 300387, China

**Keywords:** Ag_2_CO_3_ nanoparticles, biocompatibility, metabolic effects, insulin, molecular docking

## Abstract

**Background/Objectives**: Metallic NPs have been explored for various therapeutic uses owing to utilitarian physicochemical characteristics such as antibacterial, anti-inflammatory, and healing properties. The objective of this study is to evaluate the therapeutic potential of novel silver carbonate nanostructures in promoting wound healing and their antibacterial activity against *Pseudomonas chengduensis* and *Staphylococcus aureus*. **Methods**: In this work, we prepared Ag_2_CO_3_ nanoparticles through a two-step methodology that was expected to improve the material’s biomedical performance and biocompatibility. The characterization and assessment of synthesized NPs biocompatibility were conducted using hemolysis assays on the blood of a healthy male human. Further, we performed molecular docking analysis to confirm interactions of silver NPs with biological molecules. **Results**: In detail, the synthesized NPs showed <5% hemolysis activity at various concentrations, confirming their therapeutic applicability. Additionally, the NPs had good metabolic activities; they raised the T3/T4 hormone content and acted effectively on Insulin-like Growth Factor 1 (IGF-1) in diabetic models. They also facilitated the rate of repair by having the diabetic wounds reach 100% re-epithelialization by day 13, unlike the control group, which reached the same level only by day 16. The synthesized Ag_2_CO_3_ NPs exhibited high antimicrobial potential against both *Pseudomonas chengduensis* and *Staphylococcus aureus,* hence being a potential material that can be used for infection control in biomedical tissue engineering applications. **Conclusions**: These findings concluded that novel synthesis methods lead to the formation of NPs with higher therapeutic prospects; however, studies of their metaphysical and endocrinological effects are necessary.

## 1. Introduction

Among all metallic nanostructures, silver has been widely investigated in recent years primarily because of its attractive physicochemical characteristics, which have been identified as ideal for various applications, particularly in the purification of water and air, as well as in the biomedical field. These nanostructures have a high surface area and, therefore, increased reactivity, which enhances their catalytic and antimicrobial properties. Among the synthesis approaches, chemical reduction methods can be considered effective for forming silver nanostructures with pre-set nano shapes and sizes [1]. This method offers good control over the synthesis, thereby producing nanostructures that demonstrate high performance in various applications [2].

Silver nanostructures also have tremendous applications in the biomedical sector, including wound healing, metabolic regulation, and antimicrobial activity. The antibacterial characteristics of AgNPs are well known, as they are effective against Gram-positive and Gram-negative bacterial and fungal pathogens, as well as viruses. This makes silver nanostructures irreplaceable in preventing infections and treating injuries [3]. These nanostructures have enlarged surfaces, allowing for a more effective interface with microbial cells, thus enhancing their antimicrobial properties [4]. Furthermore, they are biocompatible, stimulate cell growth, and facilitate tissue formation, which shortens healing time [5].

The wound healing potential of silver nanostructures is thought to stem from their ability to decrease inflammation and increase collagen synthesis. This is due to the release of silver ions, which are believed to have antibacterial and anti-inflammatory effects, especially when used in hydrogels or other wound dressings [6]. This slow release is particularly desirable when treating chronic wounds, and materials containing silver nanoparticles have been designed to release medication gradually over a long period. Research has shown that incorporating silver nanoparticles into wound dressings improves their effectiveness, especially in treating diabetic ulcers and burns [7].

Another potential application of silver nanostructures in biomedicine is metabolic activity regulation. These nanostructures can positively affect cellular metabolism, which is beneficial for therapeutic applications. For instance, silver nanoparticles have been shown to alter oxidative stress and inflammatory activity levels in cells, making them useful for treating wound infections and other related biomedical issues [8]. Modulating cellular metabolic processes through silver nanostructures creates the foundation for personalized treatments and better patient outcomes [9]. Moreover, chemical reduction methods for preparing silver nanostructures improve their environmental and biomedical prospects while also supporting sustainable development goals [10]. These methods utilize appropriate chemicals to reduce Ag ions to Ag NPs, producing higher-quality nanostructures with uniform characteristics [11]. Due to their improved activity and biocompatibility, chemically synthesized silver nanostructures have potential applications across many sectors [12].

Despite the many opportunities, there are some challenges concerning the application of silver nanostructures in environmental and biomedical fields. For example, there is a possibility of bioaccumulation and toxicity to nontarget organisms, along with concerns about nanoparticle durability in different environments and the need for long-term studies [13]. Addressing these issues will require collaboration across materials science, toxicology, and environmental science, as well as advancements in the synthesis of safe and promising silver nanostructures [14].

The increasing need for solutions to environmental pollution and the ongoing search for better biomedical treatments underscores the importance of this work. The objective of the current research is to prepare silver nanostructures using a chemical reduction process and assess their catalytic efficiencies in degrading methylene orange dye and agricultural pesticides, as well as their wound healing, metabolic, and antimicrobial properties. This research is relevant because it could lead to the development of environmentally friendly methods for pollution treatment and effective therapeutic approaches for wound and infection treatment. Enhancing the knowledge of chemically synthesized silver nanostructures through this study promotes technological growth, which will benefit society as a whole.

## 2. Results

### 2.1. Synthesis and Characterization

**Figure 1:**
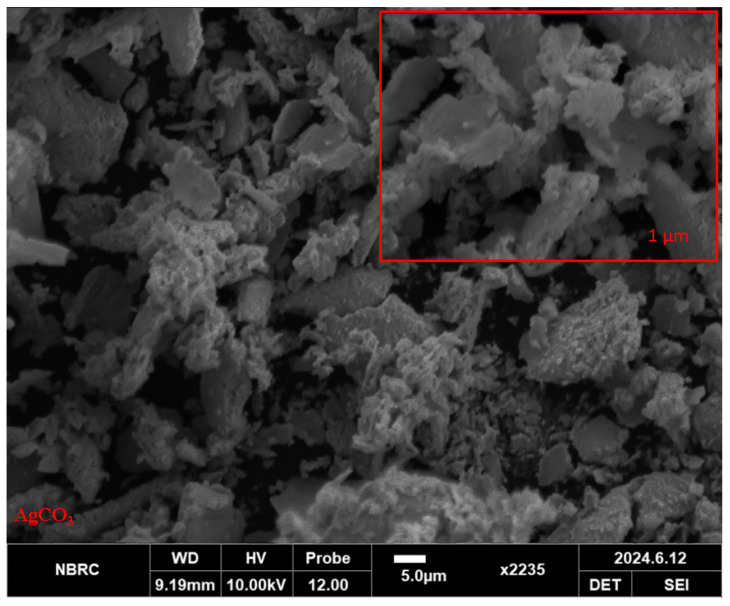
SEM analysis of Ag_2_CO_3_ nanoparticles.

**Figure 2:**
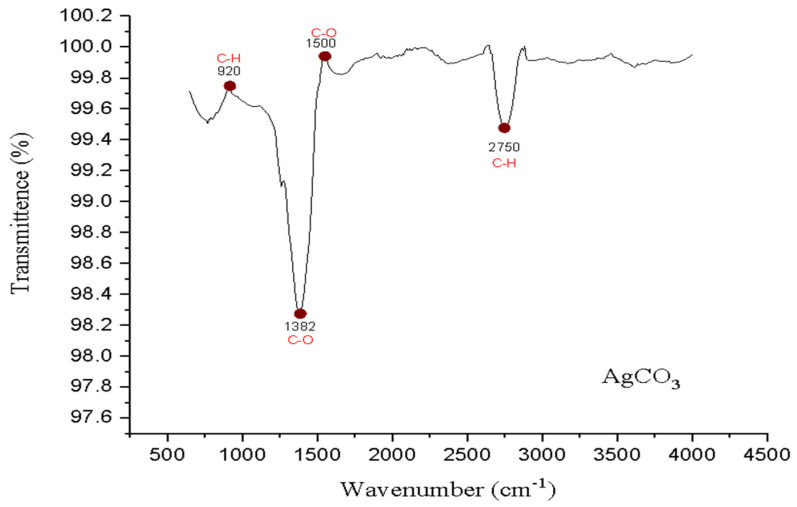
FTIR analysis of Ag_2_CO_3_ nanoparticles.

The Ag_2_CO_3_ nanoparticles were synthesized via a chemical reduction method. The characterization of these NPs was performed through SEM, FTIR, and XRD analysis. SEM analysis showed that Ag_2_CO_3_ nanoparticles have an irregular and aggregated shape with a rough and jack-surfaced morphology. These particles seem to be irregular in size and can be seen coalescing to form clusters with an impression of agglomeration. Yet, the most apparent change is related to surface texture; it is rather rough, which may affect the reactivity of the material or its uses. For such morphology, we can encounter a nonuniform synthesis process, which is characteristic of many nanoparticle fabrication techniques (Figure 1). The size of synthesized NPs was 10.29 nm (Figure 2).

In the present study, the FTIR spectrum of Ag_2_CO_3_ nanoparticles has been observed to have peaks that characterize certain functional groups. The intense characteristic peaks at 1382 cm^−1^ and 1500 cm^−1^ may be assigned to C-O for the carbonate group. The peak at 920 cm^−1^ is assigned to C-H bending, while the peak at 2750 cm^−1^ corresponds to the stretching of C-H, indicating the presence of some forms of organic residues or surface changes. These peaks further illustrate the existence of carbonate groups and other functional groups in the Ag_2_CO_3_ nanoparticles and prove the chemical composition and structure of the nanoparticles. Functional groups are indicated in red on the specified peaks identifying the functional group associated with each peak. (Figure 2).

**Figure 3:**
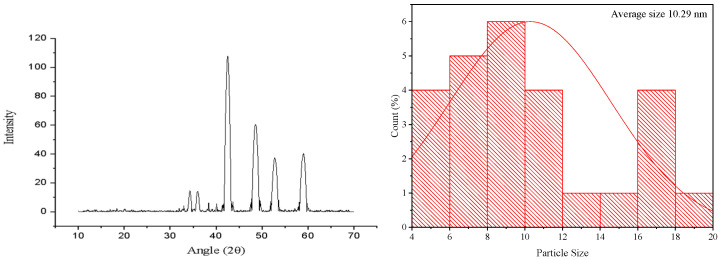
XRD analysis (**left** side) and histogram for NPs size (**right** side) of Ag_2_CO_3_ nanoparticles.

The X-ray diffraction pattern of Ag_2_CO_3_ nanoparticles shows a number of intense and sharp peaks, which confirm the crystalline behavior of the compound. The diffraction peaks at some 2θ values, evident as high-intensity peaks, belong to the characteristic crystal planes of Ag_2_CO_3_. These peaks are more intense and sharper, which indicates high crystallinity; they provide the essence of the material depending on its properties and uses (Figure 3).

### 2.2. Biocompatibility

Biocompatibility assessment in terms of hemolytic analysis showed dose-dependent hemolysis of RBCs treated with Ag_2_CO_3_ nanoparticles. At a concentration of 75 µg/mL, the level of hemolysis was observed at 3.37%. In this case, rates of hemolysis lower than 5% are generally believed to point to good biocompatibility [15]. Therefore, it is possible to conclude that the Ag_2_CO_3_ NPs could be considered biocompatible by means of red blood cells’ integrity at different concentrations. This low level of hemolysis favors the possibility of using these nanoparticles in biomedical applications and suggests that they might not pose a high degree of hemolytic toxicity in biological systems. This finding is relevant for the safe incorporation of Ag_2_CO_3_ NPs in therapeutic or diagnostic applications (Table 1).

### 2.3. Metabolic Effects

**Figure 4:**
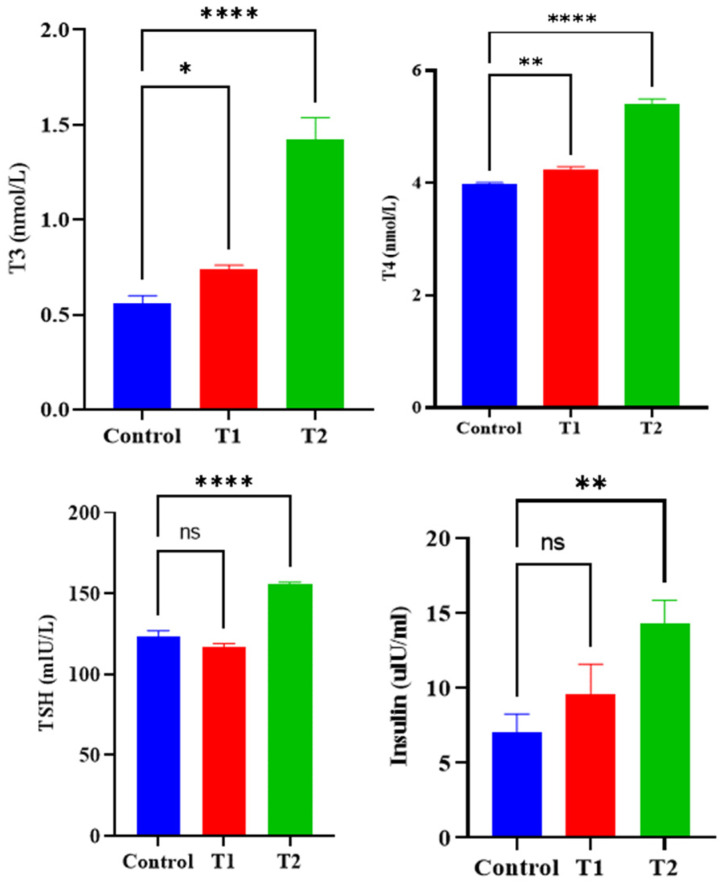
Effect of Ag_2_CO_3_ nanoparticles on metabolic profile of diabetic albino mice. *: *p* < 0.05—indicates a significant difference. **: *p* < 0.01—indicates a very significant difference. ****: *p* < 0.0001—indicates an extremely significant difference. ns: *p* > 0.05—indicates non-significant difference.

Results of metabolic investigations showed that Ag_2_CO_3_ nanoparticles can alter the metabolic pattern of diabetic albino mice in a dose-dependent manner. The high dose of NPs (50 mg/kg) significantly increased the levels of T3, T4, TSH, and insulin, which may suggest a Ag_2_CO_3_-induced stimulatory effect on thyroid hormone production that was not observed at the low dose. Thus, this study brings to light the multifaceted relationship between Ag_2_CO_3_ NPs and endocrinal functions, which suggests the need to understand the nature of any therapeutic or deleterious impact of these NPs in diabetes (Figure 4).

### 2.4. Wound Healing

**Figure 5:**
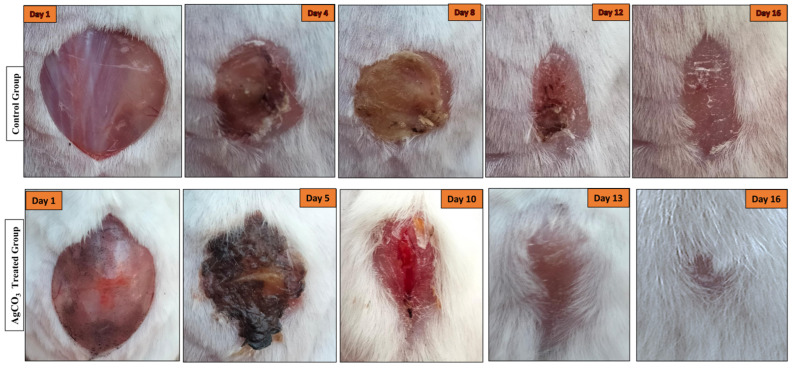
Comparison of wound healing progress between control and Ag_2_CO_3_-nanoparticle-treated diabetic wounds.

**Figure 6:**
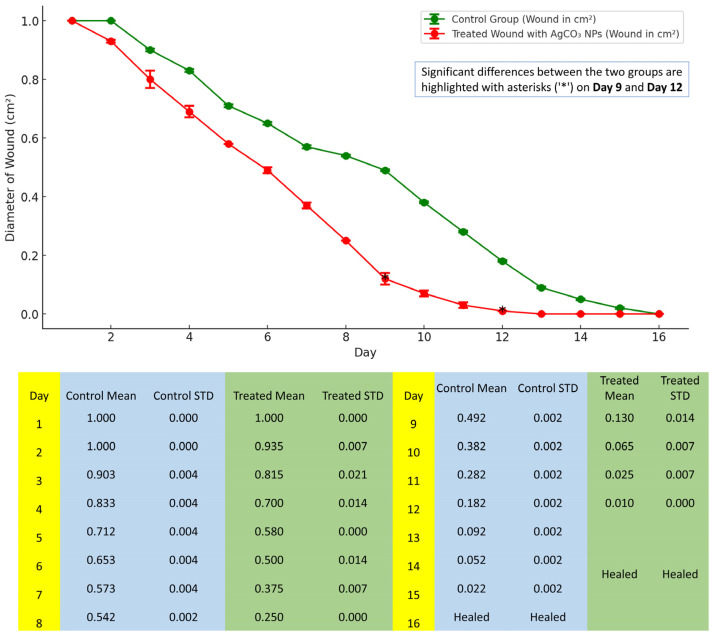
Graphical representation of wound healing progress between control and Ag_2_CO_3_-nanoparticle-treated diabetic wounds.

The data indicate that wounds treated with Ag_2_CO_3_ nanoparticles exhibit a significantly faster healing rate compared to the control group. Starting from day 2, the treated wounds show a more rapid reduction in wound size, with the healing process accelerating notably from day 5 onwards. By day 12, the treated wounds are nearly healed and, by day 13, complete healing is achieved. In contrast, the control group continues to show gradual wound closure, with complete healing not occurring until day 16. These results suggest that Ag_2_CO_3_ nanoparticles effectively enhance the wound healing process in diabetic conditions, potentially due to their antimicrobial properties and ability to promote tissue regeneration. This accelerated healing in the treated group highlights the potential therapeutic benefits of Ag_2_CO_3_ NPs in managing diabetic wounds, which are typically challenging to treat due to impaired healing mechanisms in diabetic patients (Figure 5 and Figure 6).

### 2.5. Antimicrobial Activity

**Figure 7:**
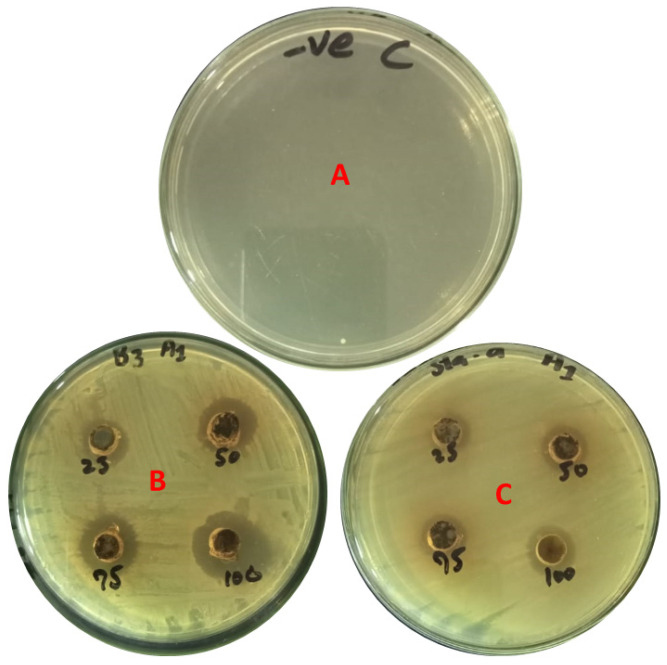
Antimicrobial activity of Ag_2_CO_3_ nanoparticle against different bacterial strains: (**A**) Control; (**B**) *Pseudomonas chengduensis*; (**C**) Staphylococcus *aureus*.

According to the data, 1 mg/mL Ag_2_CO_3_ solution has a bacteriostatic effect on both *P. chengduensis* and *S. aureus*, with the zone of inhibition growing with the increase in volume of the tested solution. *Pseudomonas chengduensis* is more sensitive to Ag_2_CO_3_ at lower concentrations (25 µL and 50 µL), as there were larger zones of inhibition at lower concentrations. Nevertheless, the inhibition seen at 75 µL and 100 µL is comparable and, in this case, it can be postulated that increasing the volume leads to increased inhibition of both bacteria by Ag_2_CO_3_ because of the accelerated antimicrobial effect of the compound. These results indicate that Ag_2_CO_3_ has pronounced broad-spectrum antimicrobial activity, with a slight tendency towards higher effectiveness than *Pseudomonas chengduensis* at high concentrations (Table 2; Figure 7).

### 2.6. Conformation of Interaction Between Ag_2_CO_3_ and Biological Proteins Involved in Metabolism Regulation and Antimicrobial Activity Through Molecular Docking Analysis

#### 2.6.1. Molecular Interaction Between Ag_2_CO_3_ and Thyroxine (T4) (PDB: 2CEO)

The molecular docking analysis presented highlights the interactions between Ag_2_CO_3_ and the thyroxine-binding protein (PDB). Records from this study portrayed the gene as 2CEO, which is known to have an important function in metabolism. The schemes at the left represent 2D depictions of the interaction between Ag_2_CO_3_ and the key amino acid residues for binding to thyroxine. Green-shaded residues, such as ALA256, ILE198, LEU261, PHE258, and TYR262, represent good van der Waals interactions that are crucial for the stabilization of the ligand in the active site and, thus, they adopted potential modulation of metabolic rate. Nonetheless, the residues colored in red (ARG260, GLU259, and PHE165) define steric or spatial hindrances expressed as “bad contacts” or “unfavorable bumps” that, potentially, lower the binding ability of Ag_2_CO_3_.

**Figure 8:**
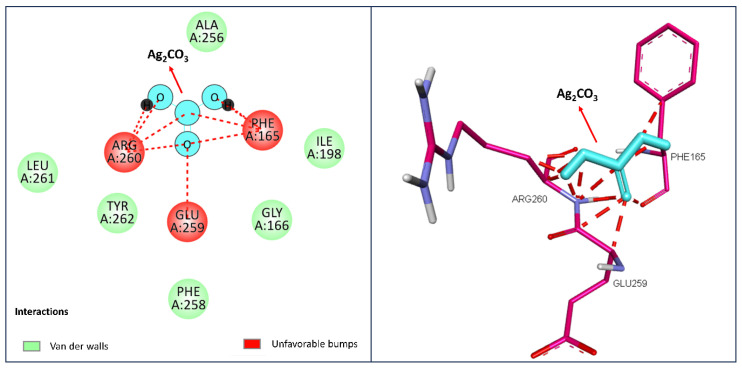
Molecular docking analysis of Ag_2_CO_3_ with thyroxine-binding protein (PDB: 2CEO): left-side figure shows 2D interactions, while right panel displays 3D binding pose.

From the 3D diagram on the right, the spatial position of Ag_2_CO_3_ within the binding site is described in more detail. The red dotted lines show that the interactions are unfavorable with Arg260, Glu 259, and Phe165 residues in particular. Such clashes may imply that, in order to obtain a better binding fit for Ag_2_CO_3_, changes may need to be made structurally so that the compound may be able to occupy a more relevant position in its interaction with the protein more effectively. This means that the observed Glide XP GScore of −3.52 indicates that there is moderate binding affinity; it shows that, while forming a stable complex with thyroxine-binding protein, there is still an area of opportunity for improvement in the binding of Ag_2_CO_3_. Since thyroxine controls metabolic processes and Ag_2_CO_3_ is involved in binding pocket, it might disturb protein conformation or flexibility and therefore disturb its role in the management of metabolic rates. Ag_2_CO_3_ could either augment or suppress all or some of the metabolic regulatory functions of thyroxine depending on the change in the overall conformation and activity of the protein induced by the ligand binding (Figure 8).

**Figure 9:**
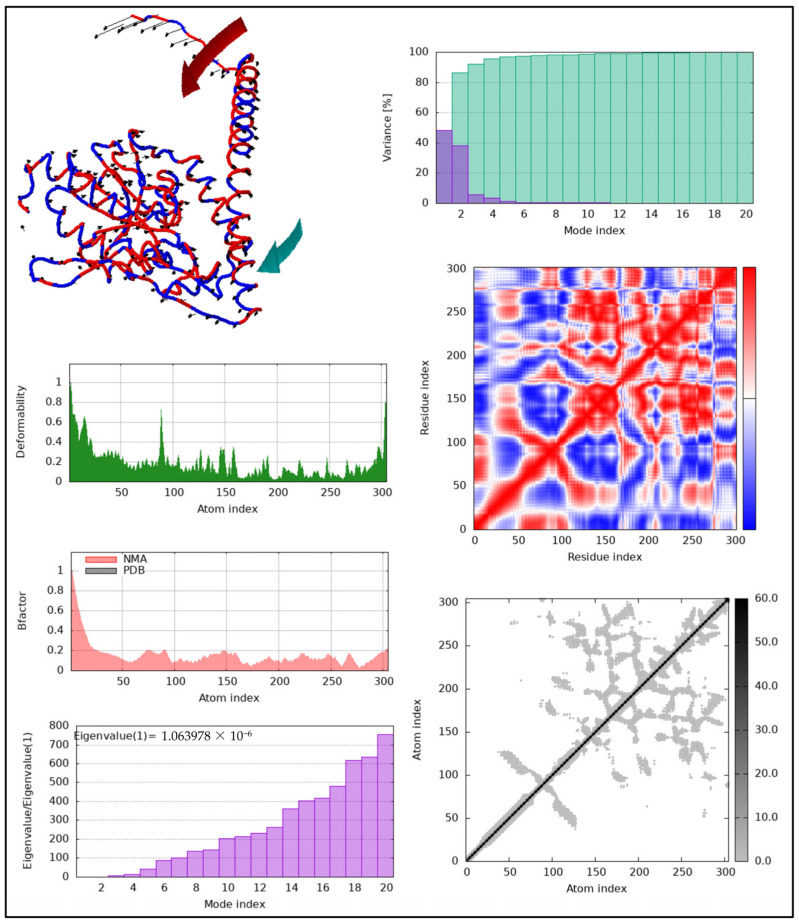
iMODS validation of thyroxine–Ag_2_CO_3_ complex: analysis of protein flexibility, deformability, and motion through normal mode analysis (NMA), highlighting dynamic conformational changes and stable interactions in the docked structure.

Figure 9 shows an iMODS validation chart related to the docking analysis, where the major interest is on the protein–ligand complex motion. Normal mode analysis used in the work is proved by the arrows in the top-left diagram, where the location of protein motion is described. This facilitates visualizing flexibility in the protein structure during the interaction process. The deformability graph, in the bottom left of the figure, represents areas of protein that are capable of changing their structures, as identified by peaks. The B-factor plot (middle left) is a graph of NMA-predicted flexibility (red line) against the flexibility as obtained from the PDB (grey bar plot), and alignment of the peaks on the two graphs indicates that the flexibility calculated by the NMA simulation was correct.

Based on the eigenvalue plot (top right), it is low (1.06 × 10^−6^), representing flexibility of the system and the ability to undergo conformational changes upon ligand binding. The variance graph (bottom right) indicates that flexibility and lower modes account for much of the motion, and higher modes have less influence in the system. The covariance matrix in the middle right shows red for correlated motions and blue for anti-correlated motions between residues. Lastly, there is the elastic network model (bottom right) that relates the forces between atoms, where the black areas indicate strong interactions diagonally along the protein structure. In summary, to the validation of the iMODS results, there is flexibility of the protein–ligand complex, implying that the protein changes its conformation in order to interact with Ag_2_CO_3_; this is a key means through which the protein is able to maintain the kinetic control of the metabolic system.

#### 2.6.2. Molecular Interaction Between Ag_2_CO_3_ and Thymidylate Kinase (PDB ID: 4GQQ)

The molecular docking analysis between Ag_2_CO_3_ and thymidylate kinase (PDB: Protein P4GQQ) has functional domains that suggest interactions critical to understanding its function in the antimicrobial property. The 2D interaction diagram (left) also depicts no covalent link between the enzyme and the inhibitor; however, Ag_2_CO_3_ has a conventional hydrogen bond with GLU161 with a green dashed line, meaning that there is a stabilizing contribution from this source to the inhibition of the enzyme. Nevertheless, residues GLU165, ILE164, and VAL163 exhibited steric clashes, and the introduction of red dashed lines indicates that this is actually a detrimental factor that may weaken Ag_2_CO_3_ binding because there may be spatial hindrance.

In the case of the 3D diagram (right), spatial positioning of Ag_2_CO_3_ is shown, especially its hydrogen bonding interaction with the GLU161 residue. However, several favorable interactions with nearby residues include the induction of six hydrogen bonds with CDR-H loop residues; however, steric clashes were also observed with nearby residues such as ILE 164 and GLU 165, which could have been minimized perfectly. At an XP GScore of −3.55, it can be argued that the binding affinity of Ag_2_CO_3_ is moderate to thymidylate kinase, and better ligand optimization can be performed to minimize steric hindrance.

**Figure 10:**
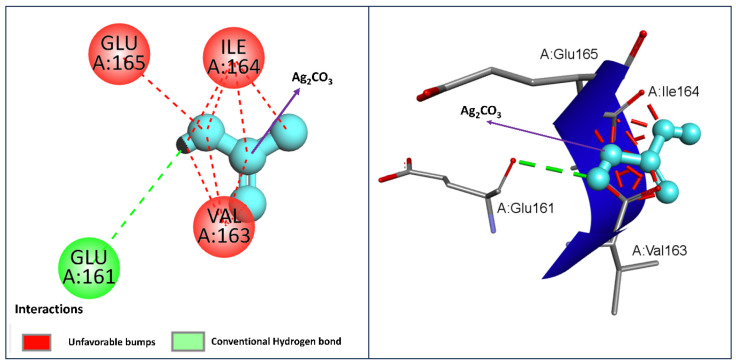
Molecular docking analysis of Ag_2_CO_3_ with thymidylate kinase (PDB ID: 4GQQ): left-side figure shows 2D interactions, while right panel displays 3D binding pose.

Thymidylate kinase is involved in DNA replication as well as the DNA repair process in microorganisms; hence, it is highly probable that Ag_2_CO_3_ acting as an inhibitor binds on the active site of the enzyme and prevents its proper working. This may inhibit microbial DNA replication, which could also account for the compound’s antibacterial properties. Thus, the analysis of docking suggests that Ag_2_CO_3_ can be used as an antimicrobial agent through interaction with the important enzyme of microbial reproduction, though further enhancement in binding energy could be conducted by structural modification (Figure 10).

This presents the iMODS validation of the thymidylate kinase–Ag_2_CO_3_, including the protein’s flexibility, deformability, and dynamic movement derived from normal mode analysis (NMA). The various figures provide a clear description of the stability of the complex and its interactivity behavior. The top left structure shows the whole conformation change of the protein using arrows to represent the direction of the flexibility for each region of the structure to illustrate the collective motions of the protein in the NMA. The deformability plot (bottom left) shows peaks that are in line with the regions where higher flexibility is confirmed, along with specific residues that indicate areas more sensitive to conformational changes.

The B-factor plot (middle left) overlays the experimental B-factor derived from the PDB with the corresponding predicted values in NMA, and the closeness of the graphs about the same axis reflects the correlation between the experimental and computational data, further affirming the docking outcomes. The variance and eigenvalue plots at the top right and bottom left prove the contribution of each mode to the overall movement, and lower modes are highly significant in overall conformational changes.

**Figure 11:**
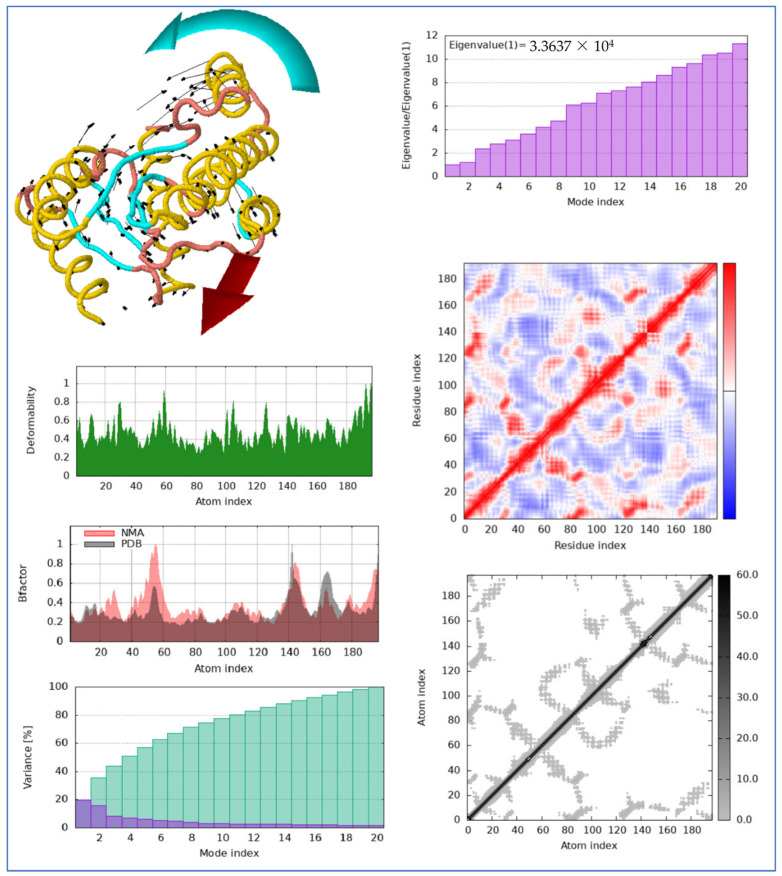
iMODS validation of thymidylate kinase–Ag_2_CO_3_ complex: analysis of protein flexibility, deformability, and motion through normal mode analysis (NMA), highlighting dynamic conformational changes and stable interactions in the docked structure.

The heatmaps (right side) also give the residue–residue contact analysis, which shows how various regions of the protein change position relative to another. The colored correlation matrix in the middle right shows an overall co-operation in motion, while the grayscale in the bottom right shows a detailed interaction of the protein. Collectively, these analyses verify the stability of the thymidylate kinase–Ag_2_CO_3_ structure and provide the cinematographic view of the protein-induced fit mechanism as to how specificity is maintained while the overall protein presents conformational flexibility (Figure 11).

## 3. Discussion

The FTIR spectrum of Ag_2_CO_3_ nanoparticles shows some intense peaks that clearly identify functional groups present in the nanoparticles. The two sharp peaks at 1382 cm^−1^ and 1500 cm^−1^ can be ascribed to C-O bonds present in the carbonate group, which is similar to findings by Pan et al. [16] in Ag_2_CO_3_ nanocomposites with photocatalytic applications. The 920 cm^−1^ peak is attributed to C-H bending and the 2750 cm^−1^ peak to C-H stretching, which implies the presence of organic residues or surface modifications, as pointed out by Rabchinskii et al. [17], where carbon–hydrogen peaks are attributed to surface changes. Furthermore, the appearance of these peaks is confirmed by Ebrahimi et al. [18], who identified Ag_2_CO_3_-based nanocomposites using FTIR, stressing the role of functional groups in the FTIR spectra of the nanoparticles. Altogether, these results confirm the presence of carbonate groups and possible surface changes in the Ag_2_CO_3_ nanoparticles.

The biocompatibility implications of Ag_2_CO_3_ nanoparticles (NPs) indicate the possibility and safety of the compound being used in biomedical applications. Hemolysis assays show the effect of concentration; the percentage of hemolysis is still below 5% at the tested range and even at the highest concentration of 75 µg/mL. Due to the low degree of hemolysis, it can be concluded that Ag_2_CO_3_ NPs provide stability to red blood cells, which is consistent with other works stating the biological compatibility of silver nanoparticles. For example, Burdușel et al. argue that, although silver nanoparticles have been shown to be toxic in high concentrations or when introduced into the human body, they are biocompatible when used in moderate quantities. This suggests that Ag_2_CO_3_ can be used in medicine, for instance, in wound dressing and infection control [5]. This is crucial, as biocompatibility must be achieved to reduce side effects, such as hemolytic toxicity, making the nanoparticles suitable for diagnosis and treatment [6].

One cannot overlook the metabolic impacts associated with Ag_2_CO_3_ NPs. The observed elevation of T3, T4, TSH, and insulin levels in experimental diabetic mice suggests that Ag_2_CO_3_ has the capability to stimulate thyroid hormone synthesis, which may directly affect global glucose homeostasis. These observations align with other studies that have shown silver nanoparticles affecting metabolic pathways. Jarak et al. further noted that the metabolic effects of silver nanoparticles include changes in various organs, indicating that the effects of Ag_2_CO_3_ on thyroid hormones are likely to have a general impact [19]. Moreover, these changes could lead to better glycemic control, which could be beneficial in managing diabetes, as seen in the study by Paul et al. [19] on the effects of silver nanoparticles on diabetes. However, the metabolic effects suggest interactive qualities, requiring further investigation to ensure that these nanoparticles do not negatively interfere with endocrine balance [20].

As to the general wound-healing abilities of Ag_2_CO_3_ NPs, the results highlight the positive impact of the developed conjugates on boosting wound closure rates, especially under diabetic conditions. The nanoparticles promoted faster wound healing, with complete closure by the 13th day, compared to the control group, which saw closure on the 16th day. These findings are consistent with previous research that highlights the positive effects of silver nanoparticles on wound healing due to their enhanced antibacterial properties and tissue remodeling function [7]. Similarly, studies by Adibhesami et al. and Bhagavathy & Kancharla indicate that silver nanoparticles significantly improve wound healing, particularly in open or diabetic wounds [21,22]. Ag_2_CO_3_ NPs are promising for wound healing applications due to their broad-spectrum antimicrobial activity and ability to promote cell proliferation in the affected area. In this study, the results obtained against *Pseudomonas chengduensis* and *Staphylococcus aureus* confirm their bacteriostatic activity, enhancing their therapeutic value in wound healing [12].

Regarding molecular interactions, data from docking studies show that Ag_2_CO_3_ NPs can affect the conformational structure of thyroxine, potentially altering thyroid hormone levels in the body. These changes could impact glucose homeostasis and insulin signaling since thyroid hormone modifications affect many metabolic processes [22]. Surprisingly, this study did not establish a direct interaction between Ag_2_CO_3_ and insulin but, given the observed effects on thyroid hormone secretion, it can be inferred that Ag_2_CO_3_ may indirectly influence insulin. This is consistent with the existing literature on thyroid dysfunction’s effects on insulin sensitivity and glucose metabolism, as reported by Sulaiman et al. [23].

The molecular docking and interaction analysis of Ag_2_CO_3_ with thymidylate kinase (PDB ID: 4GQQ) further suggest that this compound may inhibit bacterial thymidylate kinase activity. The docking results indicate that Ag_2_CO_3_ engages in both positive and negative interactions with critical amino acids essential for the enzyme’s function, particularly Glu161, Glu165, and Ile164. This interaction profile corresponds with the assertion of Jayanthi and Azam that thymidylate kinase is a prime target for new antibacterial agents [24]. Additionally, normal mode analysis (NMA) by iMODS confirmed the stability and dynamic conformational flexibility of the thymidylate kinase–Ag_2_CO_3_ complex, predicting its potential as a strong inhibitor. Khan et al. [25] also validated the effectiveness of structure-based design in identifying inhibitors targeting viral kinases, while Patel et al. [26] affirmed that computational modeling and molecular dynamics are useful in evaluating the efficacy of phytochemical inhibitors [25,26]. Altogether, these works underscore the importance of computationally guided approaches in identifying and optimizing effective thymidylate kinase inhibitors for antibacterial and antiviral applications.

## 4. Materials and Methods

### 4.1. Research Design

The research design of this study was systematically planned based on synthesizing silver carbonate nanostructures (Ag_2_CO_3_-NS) using controlled chemical reduction and characterizing them by XRD, SEM, and FTIR to confirm their structure composition, morphology, and properties, respectively. The bacterial properties of Ag_2_CO_3_-NS were evaluated by in vitro tests using different types of pathogenic bacteria. After that, the in vivo part of the study was carried out with albino mice in order to assess the metabolic performance of the nanostructures, paying attention to glucose level, lipid profile, and the general metabolic situation of the animal models.

### 4.2. Synthesis and Characterization

Silver nanostructures were synthesized using a chemical reduction method adopted from Umar et al. [27]. Silver nitrate (Sigma Aldrich; Lot# SZBF1070V; Darmstadt, Germany) was used as the precursor, while sodium borohydride (Sigma Aldrich; Darmstadt, Germany) was used as the reducing agent. There are two reaction conditions preparing two kinds of nanostructures. These synthesized nanoparticles were then confirmed using X-ray diffraction pattern (D8 advanced Burker; Karlsruhe, Germany), and scanning electron micrograph. These techniques are valuable to measure the size, shape, crystalline nature, and surface morphology of the nanostructures.

#### 4.2.1. Synthesis

The synthesis of 20 mM Ag_2_CO_3_ NPs was conducted via a two-step process adopted from Umar et al. [27]. Firstly, 15 mL of 20 mM AgNO_3_ solution was mixed with high-purity AgNO_3_ powder in about 15 mL deionized water, stirring continuously until glimmer transparent. After this, 10 mL of 20 mM Na_2_CO_3_ (Sigma Aldrich; Darmstadt, Germany) solution was dropped slowly into the above solution to form a silver carbonate precipitate. Such a dropwise addition also enabled the slow and controlled formation of silver carbonate so as to facilitate homogeneous nucleation of nanoparticles.

Once the addition of Na_2_CO_3_ was completed, there was use of an anionic reducing agent called NaBH_4_ (Sigma Aldrich; Darmstadt, Germany). This step formed the reduction process, which produced the silver carbonate nanoparticles within 10 min. However, to maintain stability in the nanoparticles, an additional 20 min of stirring were needed. During this period, the particles that had begun to grow in size acquired a fixed size and shape so that there was no agglomeration.

After nanoparticle synthesis was fully attained, the colloidal solution was centrifuged at 10,000 rpm for 15 min in order to precipitate nanoparticles from the reaction mixture. The liquid part of the suspension was removed and the collected solid nanoparticle pellet was washed with deionized water several times to eliminate residual amounts of the precursors or other products formed during the process. After washing, the nanoparticles were dried to obtain the final powdery form of silver carbonate nanoparticles, which were then used for characterizations.

#### 4.2.2. Characterization

Following the synthesis of silver carbonate nanoparticles (Ag_2_CO_3_ NPs), various characterization techniques were employed to thoroughly analyze their physical, chemical, and structural properties.

Fourier transform infrared spectroscopy (FT-IR; Cary630, Agilent Technologies, Santa Clara, CA, USA): The functional groups and chemical bonds of the nanoparticles synthesized were further identified with the help of FT-IR spectroscopy. The spectra were obtained and scanned from 4000 cm^−1^ to 400 cm^−1^ to determine the presence of the carbonate functional group, CO^3^−, and to confirm that of Ag_2_CO_3_. The characteristic diffraction peaks for carbonate ions were evidenced, and these allowed for understanding of the binding environment and chemistry of the nanoparticles.

X-ray diffraction (XRD): Structural analysis was conducted by XRD to establish the crystallite structure, phase composition, and size of the synthesized Ag_2_CO_3_ NPs. The analysis was made with an X-ray source Cu-Kα radiation Fizeau (λ = 1.5406 Å) in a range of 2θ = 10–80. The diffraction patterns were compared with standard reference patterns to ensure the obtained silver carbonate phase. The broadening of the peaks was used in conjunction with Scherrer’s equation to determine the average crystallite size of the nanoparticles produced.

Scanning electron microscopy (SEM): The studies of surface morphology and spatial distribution of nanoparticles were carried out using SEM (JSM-IT100; JEOL Ltd., Tokyo, Japan). High protocols of SEM gave details of particles that included shape, surface morphology, and aspects of agglomeration. The SEM analysis supported the earlier observation and conclusion that the nanoparticles synthesized were uniformly distributed, with almost equal particle size, which were usually spherical or irregular.

### 4.3. Research Animals

Albino mice were used as experimental animals. Healthy adult albino mice (*n* = 18–20) with an average age of 8 ± 1 week and a weight of 30 ± 5 g were purchased from Animal House University of Punjab, Lahore, Pakistan. Mice were kept in a controlled environment, both in terms of temperature and humidity, in plastic cages (temperature: 25.2 °C, humidity: 55%, and light/dark cycles: 12 h). A regular chow meal with 21% protein, 48.8% carbohydrate, and 3% fat were fed to the animals. The mice were acclimatized for a week before the trial. This experimental study was conducted in the Animal House, Department of Zoology, University of Okara.

#### 4.3.1. Diabetes Induction

Diabetes was induced in albino mice using alloxan, a compound known to selectively destroy pancreatic beta cells. The mice were administered a single intraperitoneal dose of 150 mg/kg of alloxan, which generated reactive oxygen species, leading to oxidative stress and the destruction of beta cells. This resulted in a significant decrease in insulin production, causing hyperglycemia and mimicking type 1 diabetes. The induced diabetic state allowed for further study of diabetes-related mechanisms and potential treatments.

#### 4.3.2. Blood Sampling and Euthanasia Procedure

The blood samples of albino mice were collected through a noninvasive technique to ensure the well-being of albino mice. Blood can be collected from several sites of albino mice [28], i.e., the lateral tail vein [29], the saphenous vein [30], the retroorbital sinus, and the cardiac puncher. In the current study, blood samples were collected from the lateral tail vein due to its accessibility and relatively low stress induced in albino mice. The vein was warmed to dilate the veins just before collecting the blood in the required amount [28]. To prevent blood clotting and contamination, blood was collected for metabolic profiling. At the end of the experiment, no death was observed.

##### Metabolic Evaluation

The concentration of circulating T3, T4, TSH, and insulin was estimated using Enzyme-linked Immunosorbent Assay (ELISA) kits (Sigma-Aldrich (Merck) Darmstadt, Germany). These hormone levels were then compared between the treated groups and the control in order to compare the metabolic loading of the treatment. The hormone scores were compared between the groups to assess if there was a significant difference.

#### 4.3.3. Hemolysis

The hemolysis assay was performed using blood collected from a 27-year-old male with a B-positive blood group. The blood was drawn into EDTA-coated tubes to prevent clotting. Red blood cells (RBCs) were isolated by washing the blood three times with phosphate-buffered saline (PBS) and centrifuging at 1500 rpm for 5 min after each wash. The washed RBCs were then resuspended in PBS to create a 2% erythrocyte suspension. To assess hemolysis, 100 µL of the RBC suspension was added to test tubes containing 100 µL of either the test compound, distilled water (positive control), or PBS (negative control). The samples were incubated at 37 °C for 1 h. After incubation, the tubes were centrifuged again to pellet any remaining intact RBCs, and the supernatant was collected. The degree of hemolysis was quantified by measuring the absorbance of the supernatant at 540 nm using a spectrophotometer. Percent hemolysis was calculated by comparing the absorbance of the experimental samples to the positive and negative controls. This procedure allowed for the assessment of the hemolytic potential of the compounds tested on human RBCs.

$$Percent\;Hemolysis=\left(\frac{Absorbance\;of\;sample-Absorbance\;of\;negative\;control}{Absorbance\;of\;positive\;control-Absorbance\;of\;negative\;control}\right)\times 100$$

### 4.4. Wound Healing

To determine the ability of the NPs in wound healing, two full-thickness standardized punched excisions (1 cm^2^) were made on the back of each albino mouse. The mice were divided into two groups, which included a control group injected with saline and an experimental group receiving silver nanostructures. The nanostructures were used locally on the wound areas once daily. The wound healing procedure was followed and assayed from digital photographic records. To find the rate of wound closure, the wound size was determined at different time points [31].

### 4.5. Antimicrobial Activity

An investigation of the synthesized silver nanostructures against pathogenic bacteria such as *Staphylococcus aureus* and Escherichia coli was also evaluated. The well diffusion method was followed, in which bacterial cultures were streaked on agar plates and wells were made to inoculate the nanostructures. The plates were incubated at 37 °C for 24 h, after which the zone of inhibition around each well was checked to determine probabilities of the antimicrobial activities.

### 4.6. Docking Study

For the docking analysis, two proteins were selected: thyroxine-binding protein (PDB ID: 2CEO), which plays a role in metabolism regulation, and thymidylate kinase (PDB ID: 4GQQ), with antimicrobial activity. Both proteins were prepared using the Protein Preparation Wizard in Maestro 12.5 software. This comprised of reading in the PDB structures, appraisal of the bonds, inclusion of hydrogen atoms, and addressing of missing side chains and loops that were missing through the PMV Prime module. In addition, hydrogen bonds were adjusted, and the structures’ geometry was fully optimized with the help of the OPLS3e force field. Additional water molecules that do not play a role with ligand interactions were clipped off from the protein structure.

Ligands were first built with the help of the LigPrep module of Maestro. This was aided by geometry optimization and energy minimization apart from tautomeric and ionization states at a pH of 7.0 ± 2.0. The structures of the ligands used in the analysis were chosen from the conformations exhibiting the least energy in their configurational space. Receptor grids were created for each protein using the Receptor Grid Generation option in the Maestro suite. This specific grid establishes the docking area and, therefore, ligands bind within the defined active site. The grid for 2CEO was constructed based on the thyroxine-binding pocket and the thymidylate kinase active site for 4GQQ. Certain modifications were made to the Van der Waals radii of all nonpolar atoms in order to achieve better flexibility within the docking space.

The ligands were then placed into their respective receptor grids using the Glide docking tool of the Maestro application. There, depending on the level of accuracy demanded, either the single-part precision docking (SP) or the double-part extra-precision docking (XP) technique was used. What was found was that the docking process provided flexible ligand sampling, which means that various conformations of the ligand can be sampled. Evaluation was conducted using the Glide Score function that takes into consideration hydrogen bonding, hydrophobic contacts, and shape complementarity. The most-ranked ligand–receptor complexes were solely considered for further analysis.

Subsequently, the protein–ligand interactions were analyzed; although, docking the protein, the interacting energies were analyzed, which include hydrogen bonds, π-π stacking, and hydrophobic interactions. Following each identified ligand, multiple poses were scrutinized to set the correct position of the ligand for binding. Finally, the docked complexes were summarized and viewed in Discovery Studio (BIOVIA Discovery Studio Client 2024). With Discovery Studio tools, detailed 2D and 3D diagrams were prepared that illustrated the interactions with and between ligands and protein residues, such as hydrogen bonds, hydrophobic pockets, and π-pi stacks. The visualization aided in the continuation of the docking results and their validation [32].

### 4.7. Statistical Analysis

All experimental data were analyzed using the statistical software (GraphPad prism v 10). The data were summarized and presented using mean ± standard deviation (SD) on the value of the variables. One-way analysis of variance (ANOVA) was used to compare the results of the control and the treatment groups. The p-value is desirable and significant, with a level usually below 0.05 being viewed as statistically meaningful.

## 5. Conclusions

In conclusion, this study explores that, by employing silver carbonate (Ag_2_CO_3_) NPs, there are tremendous opportunities to use them in various biomedical applications. The new morphology of the synthesized Ag_2_CO_3_ NPs with irregular and aggregated shapes provides better activity on the catalyst support. This was further supported by biocompatibility data showing their low hemolytic potential for safe use in therapeutic systems. There was further proof that Ag_2_CO_3_ NPs could exert metabolic effects and therefore may have endocrine-related potential benefits or risks. Moreover, the wound healing experience revealed enhanced diabetic healing involving the use of Ag_2_CO_3_ NPs effectively enhancing tissue repair. Our results indicate that they have a wide spectrum of antibacterial action against *Pseudomonas chengduensis* and *Staphylococcus aureus* and, hence, could be used as potent inhibitors against microbial infections via influencing microbial enzymes such as thymidylate kinase. Molecular docking and iMODS reaffirmed the solidity in structure and functionality of Ag_2_CO_3_ on metabolic and antimicrobial interfaces. Taken together, these works reveal that Ag_2_CO_3_ NPs have numerous potential uses in the therapeutic domain, ranging from the management of wounds to antimicrobial treatments and metabolic conditions

## Figures and Tables

**Table 1:** Hemolytic activity of different concentrations of Ag_2_CO_3_ NPs.

Sr No	Concentration (μg/mL)	Hemolysis %	F Value	*p* Value
1	25	2.55 ± 0.041	408.8	<0.0001 ***
2	50	2.98 ± 0.033		
3	75	3.37 ± 0.035		

*** = Highly significant.

**Table 2:** Antimicrobial activity (zone of inhibition) of Ag NPs.

Concentration of Ag_2_CO_3_ (1 mg/mL)	Zone of Inhibition (mm)		T Value	*p* Value
	*Pseudomonas chengduensis*	*Staphylococcus* *aureus*		
25 µL	6.52 ± 0.49	4.375 ± 0.38	18.65	0.0341 *
50 µL	7.255 ± 0.19	5.865 ± 0.27	3.022	0.2035
75 µL	7.86 ± 0.26	7.76 ± 0.26	21.0	0.0303 *
100 µL	8.405 ± 0.29	8.175 ± 0.275	23.0	0.0277 *

*: *p* < 0.05—indicates a significant difference.

## Data Availability

All the data are available in the manuscript.
